# Correction: Loading-Induced Heat-Shock Response in Bovine Intervertebral Disc Organ Culture

**DOI:** 10.1371/journal.pone.0167406

**Published:** 2016-11-22

**Authors:** Wai Hon Chooi, Samantha Chun Wai Chan, Benjamin Gantenbein, Barbara Pui Chan

## Notice of Republication

This article was republished on November 4, 2016, to correct errors in the order of the figures. The authors apologize for the errors. Please download this article again to view the correct version. The originally published, uncorrected article and the republished, corrected article are provided here for reference.

## Supporting Information

S1 File Originally published, uncorrected article. (PDF)

S2 File Republished, corrected article. (PDF)
