# Supplementary figures and images for: Loading-Induced Heat-Shock Response in Bovine Intervertebral Disc Organ Culture
Source: PLoS One. 2016 Aug 31;11(8):e0161615. doi: 10.1371/journal.pone.0161615 (PMC5006975; doi:10.1371/journal.pone.0161615)

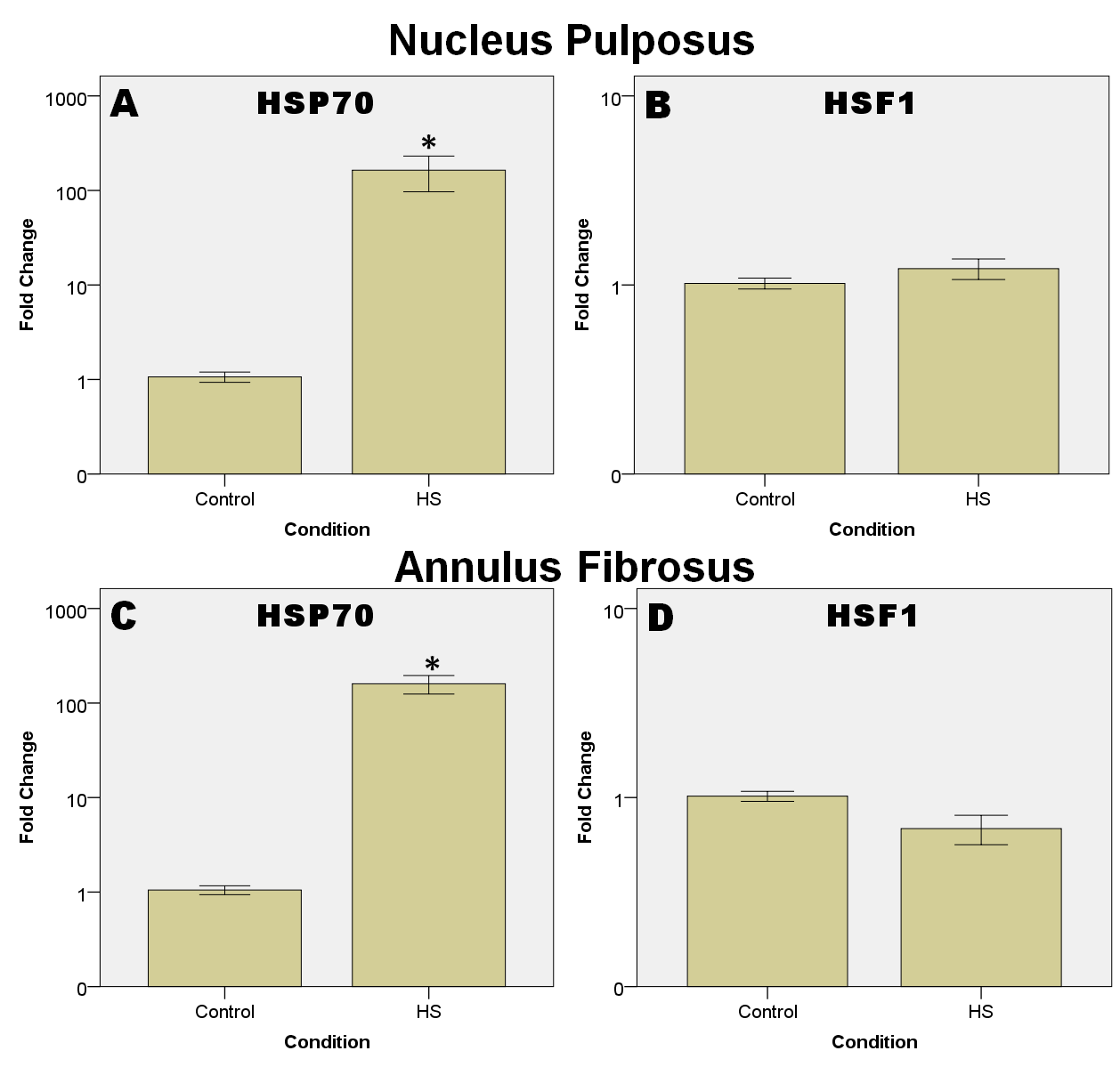

Supplement: S1 Fig — Gene expressions are in log of fold change normalized to control without loading and represented in mean±SEM. HS: Heat-Shock. * = statistical significant difference with p<0.05 compared to control. N = 5. (TIF) [file pone.0161615.s001.tif]

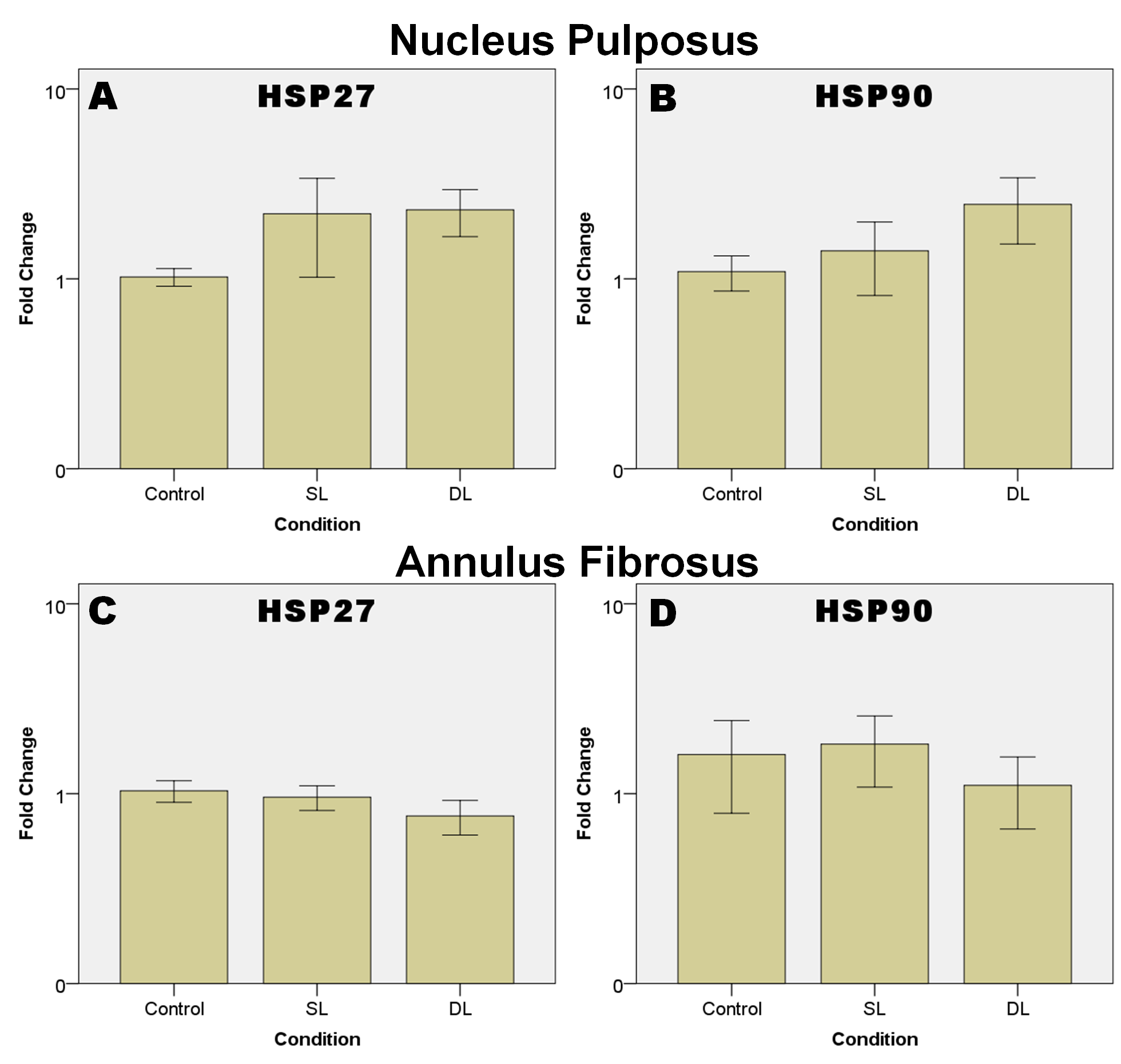

Supplement: S2 Fig — Gene expressions are in log of fold change normalized to control without loading and represented in means ± SEM. SL: Static Loading, DL: Dynamic Loading. N = 5 (from 3 animals) compared to control. (TIF) [file pone.0161615.s002.tif]
