# Supplementary material for: Correction: Loading-Induced Heat-Shock Response in Bovine Intervertebral Disc Organ Culture
Source: PLoS One. 2016 Nov 22;11(11):e0167406. doi: 10.1371/journal.pone.0167406 (PMC5119852; doi:10.1371/journal.pone.0167406)
Supplement: S1 File — (PDF) [file pone.0167406.s001.PDF]

PLOS ONE

RESEARCH ARTICLE

Loading-Induced Heat-Shock Response in Bovine Intervertebral Disc Organ Culture

Wai Hon Chooi[1], **Samantha Chun Wai Chan**[2,3], **Benjamin Gantenbein**[2], **Barbara Pui Chan**[1]*

1 Tissue Engineering Laboratory, Department of Mechanical Engineering, The University of Hong Kong, Hong Kong Special Administrative Region, China, **2** Tissue & Organ Mechanobiology, Institute of Surgical Technology and Biomechanics, University of Bern, Bern, Switzerland, **3** Biointerfaces, Empa, Swiss Federal Laboratories for Materials Science and Technology, St Gallen, Switzerland

* bpchan@hku.hk

CrossMark
click for updates

OPEN ACCESS

Citation: Chooi WH, Chan SCW, Gantenbein B, Chan BP (2016) Loading-Induced Heat-Shock Response in Bovine Intervertebral Disc Organ Culture. PLoS ONE 11(8): e0161615. doi:10.1371/journal.pone.0161615

Editor: Marc Muller, Universite de Liege, BELGIUM

Received: April 15, 2016

Accepted: August 9, 2016

Published: August 31, 2016

Copyright: © 2016 Chooi et al. This is an open access article distributed under the terms of the Creative Commons Attribution License, which permits unrestricted use, distribution, and reproduction in any medium, provided the original author and source are credited.

Data Availability Statement: All relevant data are within the paper and its Supporting Information files.

Funding: This study was partially supported by an AOSpine award (SRN_2011_14) (https://aospine.aofoundation.org/Structure/Pages/default.aspx) and a Theme-Based Research Scheme (T12-708/12-N) (http://www.ugc.edu.hk/eng/rgc/theme/theme.htm) to BP, an AOSpine Spine Research Network Grant to WH (https://aospine.aofoundation.org/Structure/Pages/default.aspx), and a Swiss National Science Foundation Project (#310030_153411) (http://www.snf.ch/en/funding/projects/Pages/default.aspx) to BG. The funders had no role in study design, data

Abstract

Mechanical loading has been shown to affect cell viability and matrix maintenance in the intervertebral disc (IVD) but there is no investigation on how cells survive mechanical stress and whether the IVD cells perceive mechanical loading as stress and respond by expression of heat shock proteins. This study investigates the stress response in the IVD in response to compressive loading. Bovine caudal disc organ culture was used to study the effect of physiological range static loading and dynamic loading. Cell activity, gene expression and immunofluorescence staining were used to analyze the cell response. Cell activity and cytoskeleton of the cells did not change significantly after loading. In gene expression analysis, significant up-regulation of heat shock protein-70 (HSP70) was observed in nucleus pulposus after two hours of loading. However, the expression of the matrix remodeling genes did not change significantly after loading. Similarly, expressions of stress response and matrix remodeling genes changed with application and removal of the dynamic loading. The results suggest that stress response was induced by physiological range loading without significantly changing cell activity and upregulating matrix remodeling. This study provides direct evidence on loading induced stress response in IVD cells and contributes to our understanding in the mechanoregulation of intervertebral disc cells.

Introduction

Degeneration of the intervertebral disc (IVD) is often associated with low back pain [1]. It affects a large number of people, reduces quality of life and causes economic loss. Studies on twins and genetics suggest that genetic factor play a major role in disc degeneration [2–4]. In addition to that, several factors have been suggested to contribute to the cause of the disease [5, 6]. Factors that are related to the patient conditions such as age and obesity as well as habit such as smoking were shown to be associated with disc degeneration [7, 8].

As IVD experience mechanical loadings during daily activities, extensive research has been done to elucidate the effects of mechanical loading on the IVD [9–12]. These studies show that high frequency and high magnitude compressive loading can cause cell death, catabolic gene

collection and analysis, decision to publish, or preparation of the manuscript.

Competing Interests: The authors have declared that no competing interests exist.

up-regulation and degeneration in IVD. However, it was unclear whether mechanical loading would cause cellular stress response in IVD.

Heat shock response is part of the cellular stress response, which acts as the cells' protection and repair mechanism when triggered by environmental stressors such as heat, ultraviolet light, toxins, pH change and oxidative stress. Mechanical loading is also demonstrated to cause similar response. It has been demonstrated that stretching could induce Heat-Shock Proteins (HSPs) upregulation in endothelial cells [13], melanocytes [14], bladder smooth muscle cells [15], vascular smooth muscle cells [16], periodontal ligament cells [17], trabecular meshwork cells [18] and tendon fibroblast [19]. Similar to the IVD, the articular cartilage is also subjected to considerable compressive loading. Expression of HSP70 was demonstrated to be up-regulated in human articular cartilage after static compression [20]. Similar findings were also reported in immortalized human chondrocytes in monolayer [21] and rabbit chondrocytes cultured in alginate beads [22] in response to high hydrostatic pressure. These results demonstrated that cells do respond to mechanical loading by upregulating HSP70.

Expression of HSPs has been shown in human IVD. Using histochemical study, HSP72 and HSP27 were found to accumulate in chondrocytes of endplate and nucleus pulposus in the IVD during childhood development but decrease with aging [23]. The percentage of HSP72 and HSP27 positive cells also increased in the degenerated IVD and HSP72 was identified specifically in the nuclei of these cells. The expressions of Heat-Shock Factor-1 (HSF1), HSP72 and HSP27 were further found to be associated with cell cluster formation and other pathological conditions such as disc herniation [24]. The occurrence of HSPs in the degenerated disc was, therefore, suggested to be associated with disc degeneration. Nevertheless, to the best of our knowledge, there is to date no study available that demonstrates that cellular stress response is directly linked to mechanical loading.

In vitro organ culture using bovine caudal disc has been established and was used as a model for mechanobiology study previously [25–28]. Similarity in bovine disc dimension and cell popularity with human disc makes it an ideal model for mechanobiology study [25–30]. Here we hypothesized that compression loading in disc organ culture models stimulates stress responses of the disc cells. In this study, we specifically aim to investigate the effects of compression loading of different types and patterns on NP and AF. Stress responses and matrix remodeling will be studied at both gene and protein level.

Materials and Methods

Tissue culture

Fresh bovine caudal discs with endplate were harvested from eight one to two years old cows and cultured according to method previously described [31]. All experimental protocols were approved by the animal research committee of the University of Bern, Switzerland and the methods were carried out in accordance with the approved guidelines. In brief, after isolating the discs from the tail, the endplates were jet-lavaged with Ringer's solution using Pulsavac wound debridement irrigation system to remove blood clots (Zimmer inc., Winterthur, Switzerland). Discs were then cultured in High glucose Dulbecco's Modified Eagle's Medium (DMEM, 4.5g/L glucose, Gibco, Life Technologies, inc., Basel, Switzerland) with 5% Fetal Calf Serum, 100 U/ml penicillin and 100 cg/ml streptomycin (all Sigma-Aldrich, Buchs, Switzerland).

Mechanical loading

As previous studies show that cell may react differently due to different loading types, cellular stress response in response to static and dynamic loading was studied. Bovine caudal discs

were loaded on to a custom made bioreactor [32] to apply compressive loading. IVD mechanobiology studies were previously performed on this bioreactor, which can maintain cell viability and nutrients diffusion throughout the experiment with dynamic compressive loads applied [30] (Fig 1). Loading pressure was calculated as force applied divided by the cross section area of the disc [30]. Compressive dynamic loading was applied at physiological range (0.35 ± 0.25 MPa, 0.2 Hz), while static loading was applied at 0.35 MPa for two h. Preliminary results

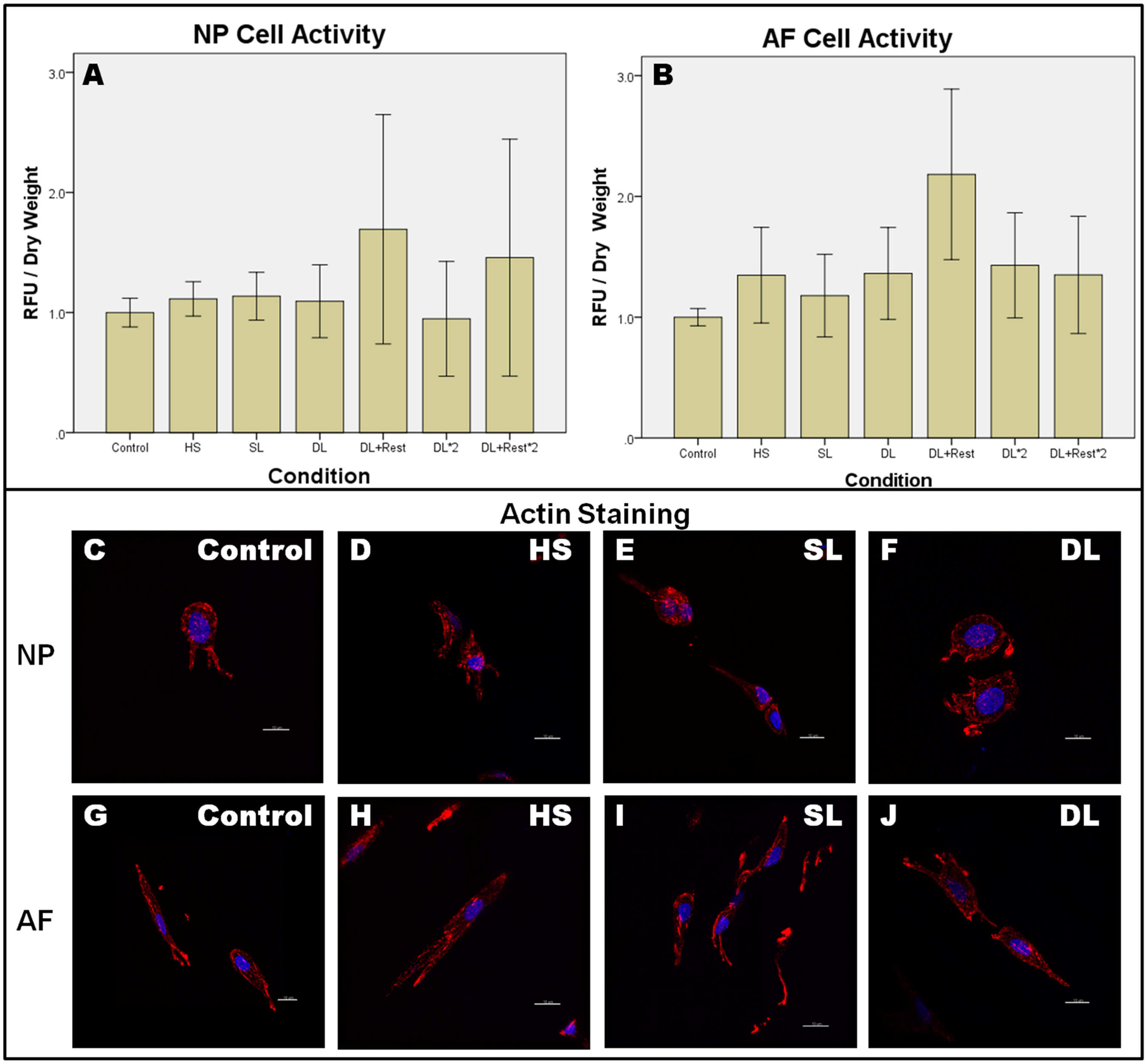

Fig 1. Customized two degree-of-freedom (i.e., compression and torsion) bioreactor to maintain disc explants alive and to apply repeated mechanical loading for two days. A. close-up view of single station harboring a bovine coccygeal IVD soaked in culture media. Biocompatible materials are enox aluminium (black), polyoxymethylene (POM, white parts) and glass with a "press-fit" design and silicon rings (black rings) to ensure no leakage between glass and POM. **B.** 4-unit design arranged in 5% CO_2 and 60% humidity incubator. **C.** Scheme of control of uniaxial compression and axial torsion using fluidic muscle and servo-controlled valve. **D.** Close-up view of serrated titanium plate surface, which grasps IVD and keeps it in place and ensures nutrition diffusion to the bony endplate.

doi:10.1371/journal.pone.0161615.g001

showed that IVD cells had the highest up-regulation of HSP70 after two h of compressive loading compared to other time points. In order to study the temporal effect of the tissue after dynamic loading, cellular response after repeated loading was investigated. The discs underwent two h of dynamic loading, followed by 22 h of resting before a second round of loading-resting cycle for two days (Fig 2). Samples were retrieved at different time points: right after loading and right after resting. Discs put under Heat-shock (43°C) treatment for two h were used as positive control. Free swelling discs cultured without any treatment were used as negative controls. Discs from the same animal were assigned to each condition to minimize inter-animal variability.

Cell activity

Cell activity was measured by resazurin assay that measures the aerobic respiration of the cells to assess cell viability [33]. Nucleus pulposus (NP) tissues and Annulus fibrosus (AF) tissues from each disc were incubated in medium with 50μM resazurin sodium salt (Sigma-Aldrich, Buchs Switzerland) for five hours. The relative fluorescence unit (RFU) was then measure using a spectrophotometer (SpectraMax M5, Molecular devices, distributed by Bucher Biotec, Switzerland). RFU measured for each tissue was normalized with the dry weight of the tissue.

Relative Gene expression

Total RNA was extracted by using TRI reagent. In brief, disc tissues were pulverized into powders in liquid nitrogen and homogenized with 1 mL TRI reagent (Molecular Research Center, Cincinnati, USA). RNA extracted was then purified using GenElute Mammalian total RNA purification kit followed by DNA digestion (Both from Sigma-Aldrich, Buchs, Switzerland). Reverse transcription was carried out by using iScript cDNA Synthesis kit (Bio-Rad, Reinach, Switzerland). Oligonucleotide primers for qPCR were designed by Beacon Designer™ (Premier Biosoft inc., Palo Alto, CA) based on sequences from NCBI GenBank database. The information of the primers is presented in Table 1. Quantitative PCR was performed on an iQ5 qPCR Detection System (Bio-Rad). Every reaction consisted of 2 μL water, 5 μL iQ-SYBR Green Super mix (Bio-Rad, Cressier, Switzerland), 1 μL primers (0.25 μM) and 2 μL cDNA. All C_t values of the genes were normalized to C_t value of ribosomal 18S RNA. Fold change of the gene expression was calculated relative to the negative control group from the same animal by $2^{-\Delta\Delta Ct}$ method.

Actin and immunofluorescence staining

Tissues were fixed in freshly-prepared 4% buffered paraformadehyde, frozen and cryo-sectioned for immunofluorescence staining. Sections of 10 μm were stained with primary antibodies of mouse monoclonal anti-HSP72 (inducible form of HSP70, at 1:100 dilution, ADI-SPA-810, Enzo, NY) and rat monoclonal anti-HSF1 (at 1:50 dilution, ab61382, Abcam, Cambridge, MA). Briefly, sections were hydrated in phosphate buffered saline solution (PBS) and permeabilized with 0.5% Tween-20 (Sigma-Aldrich, Buchs, Switzerland). Antigen was retrieved in citrate buffer (10 mM sodium citrate with 0.05% Tween-20 at pH 6.0) at 95°C for 15 min and then washed with 0.05% Tween-20. Blocking was done using 3% bovine serum albumin (Jackson Immuno Research, PA) for 30 min. Sections were then incubated with primary antibodies at 4°C for overnight. After washing with PBS, sections were then incubated in appropriate fluorochrome conjugated secondary antibodies at 1:400 dilution for 1 h. For actin staining, sections were incubated in rhodamine-phalloidin (Molecular Probes, Eugene, OR) at 1:40 dilution for one h. The sections were mounted with Fluoro-Gel II with DAPI (EMS, Hatfield, PA). Images

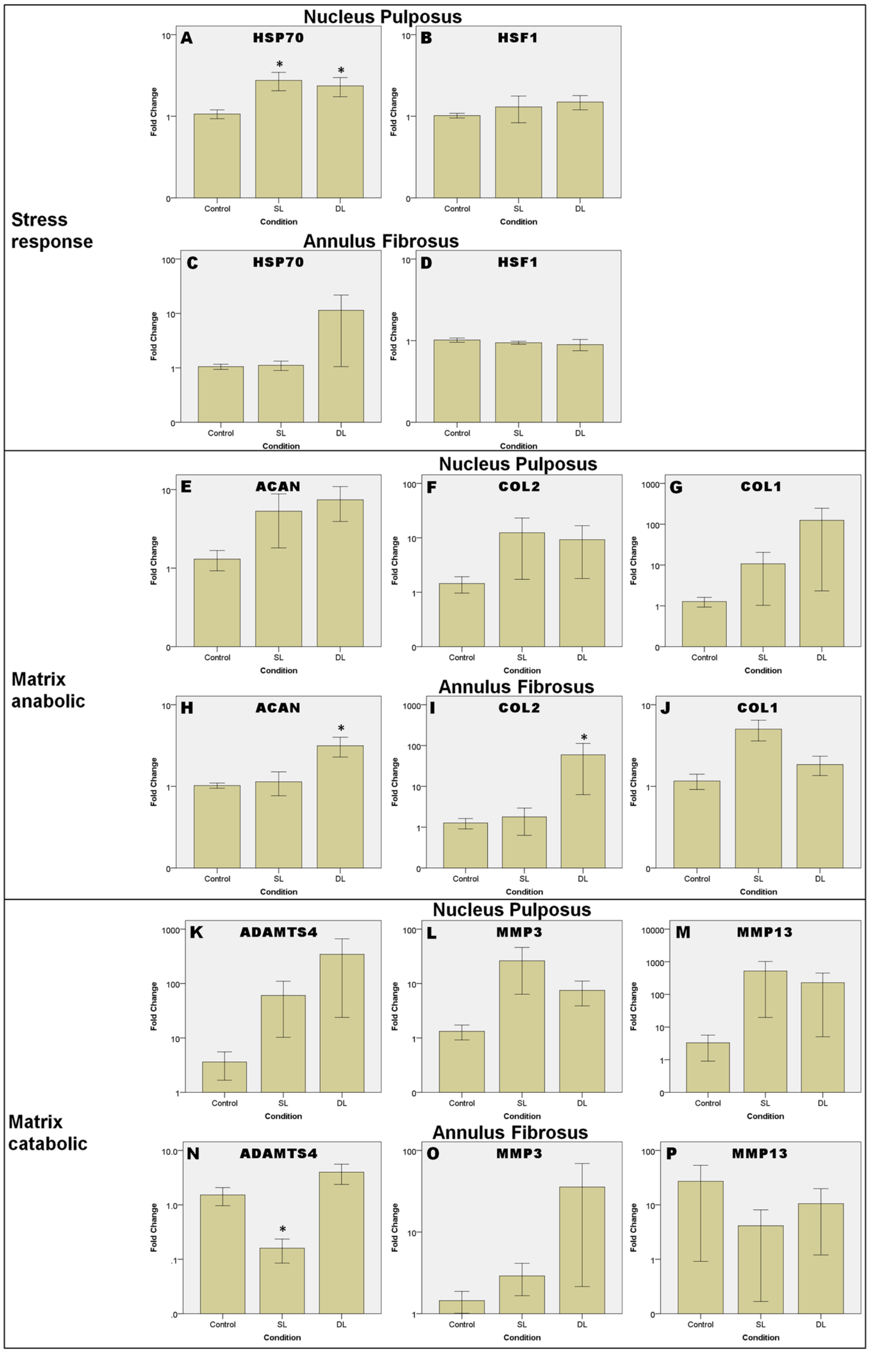

Fig 2. Conditions applied to disc explants. Heat-shock was applied on discs as positive control (A). Compressive loading of different modes was applied at physiological range 0.35 MPa (static loading) and 0.35 ± 0.25 MPa (dynamic loading) for two h (B). For repeated loading, compressive dynamic loading was for two h per day for two days (C). Samples were retrieved for analysis at different time points: 1. Control, 2. DL (right after two h of dynamic loading), 3. DL+Rest (22 h after dynamic loading), 4. DL*2 (right after two h of dynamic loading on Day two), 5. DL+Rest*2 (22 h after dynamic loading on Day two).

doi:10.1371/journal.pone.0161615.g002

were scanned using a confocal scanning microscope (LSM700, Carl Zeiss, Jena, Germany). Signal intensity of HSP70 and HSF1 in each nucleus was quantified using IMARIS software (Bitplane AG, Zurich, Switzerland) and normalized to the volume of the nucleus.

Table 1. Primers used for quantitative real time PCR in organ culture study.

Gene	Forward Primer (5' - 3')	Reverse Primer (5' - 3')
18S	ACG GAC AGG ATT GAC AGA TTG	CCA GAG TCT CGT TCG TTA TCG
HSP70	AGG AGG TGG ATT AGG AAT	GGA CAG TTC AAC ATC TCA
HSF1	GCA GGT GTT CAT AGA ATT GTA TT	CTG GCT CAT CGG TCT GTT
ACAN	GGC ATC GTG TTC CAT TAC AG	ACT CGT CCT TGT CTC CAT AG
COL2	CGG GTG AAC GTG GAG AGA CA	GTC CAG GGT TGC CAT TGG AG
COL1	GCC TCG CTC ACC AAC TTC	AGT AAC CAC TGC TCC ATT CTG
ADAMTS4	TCC TGG CTG GCT TCC TCT TC	CCT CGG ACA AGT CTT CAG AAT CTC
MMP3	CTT CCG ATT CTG CTG TTG CTA TG	ATG GTG TCT TCC TTG TCC CTT G
MMP13	TCC TGG CTG GCT TCC TCT TC	CCT CGG ACA AGT CTT CAG AAT CTC

doi:10.1371/journal.pone.0161615.t001

Statistical analysis

All statistical analyses were executed using SPSS 19.0 (IBM, NY). The significance level was set to 0.05. Non-parametric analysis of variance by rank test (Kruskal-Wallis test) was use to test significant difference between loaded groups and control. One-way ANOVA was performed to determine significant difference of cell activity among multiple groups.

Results

Cell activity and cytoskeleton

Fig 3 shows that cell activity was maintained under all conditions with no significant reduction of cell viability (One way ANOVA, $p > 0.05$). F-actin of the cells did not change substantially after heat-shock and loading with no stress fibers observed after loading.

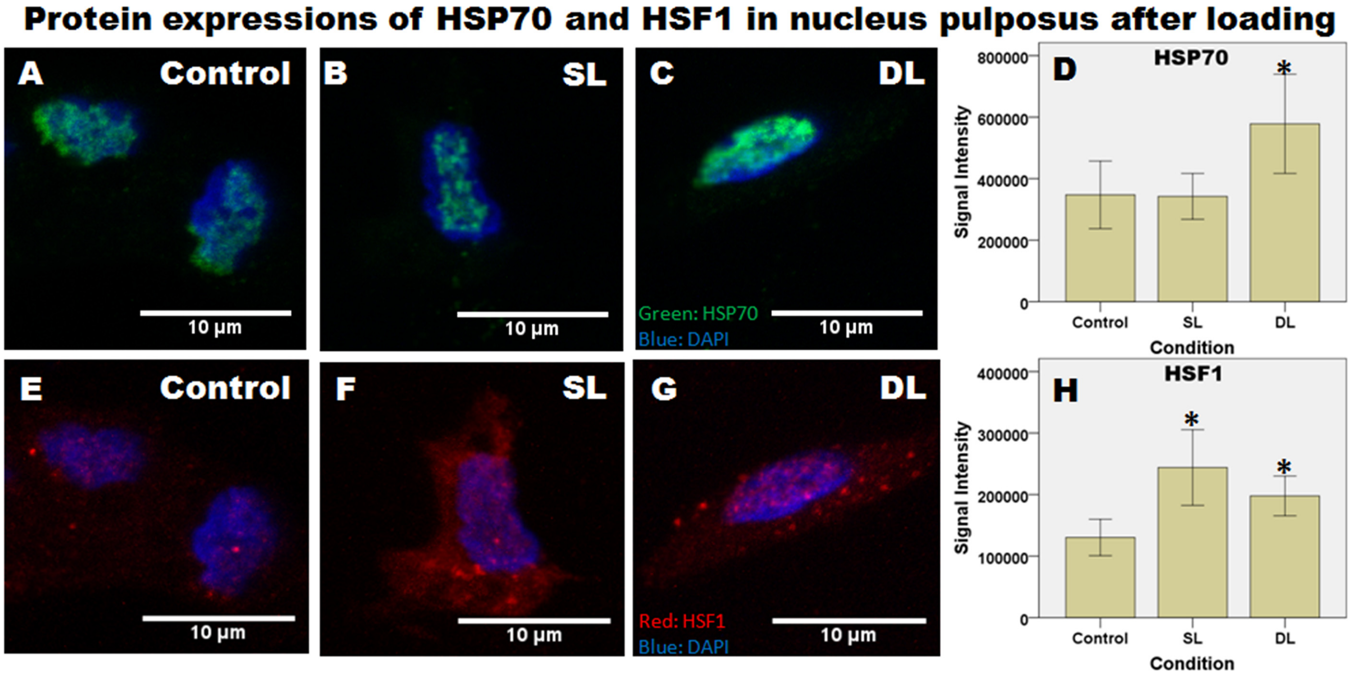

Fig 3. Cell activity and cytoskeleton were maintained after each condition. Cell activity was measured by resazurin assay (A-B) and cytoskeleton was visualized by f-actin staining (C-J) for NP and AF after each condition. Values are normalized to control without loading and represented in mean ± SEM. Control: Control discs without loading, HS: Heat-Shock, SL: Static Loading, DL: Dynamic loading, DL+Rest: Dynamic loading with Resting, DL*2: dynamic loading for two days, DL+Rest*2: dynamic loading and resting for two days. NP: Nuclues pulposus, AF: Annulus fibrosus. N = 5. RFU: relative fluorescence unit.

doi:10.1371/journal.pone.0161615.g003

Heat-shock

Heat-shock was used as the positive control to assess cells' ability to upregulate expression of HSPs. Gene expression of HSP70 and HSF1 were analyzed as both are involved in cellular response towards stresses including heat stress and mechanical stress [34, 35]. Heat-shock stress resulted in a 100-fold increase in HSP70 in both NP and AF (S1 Fig). Kruskal-Wallis test showed that the upregulation in HSP70 was statistically significant different in both NP ($p = 0.002$) and AF ($p = 0.001$) compared to negative control. On the other hand, there was no statistical significant difference in the expression of HSF1, which is the transcription factor for HSP70, HSF1 in both NP and AF.

Different loading types

Fig 4 shows the gene expressions of the tissues under different loading types under three groups of genes: stress response genes (Fig 4A–4D: HSP70 and HSF1), matrix anabolic genes (Fig 4E–4J: ACAN, COL2 and COL1) and matrix catabolic genes (Fig 4K–4P: ADAMTS4, MMP3 and MMP13).

HSP70 was up-regulated in NP after static and dynamic loading (Fig 4A) by about 2.8 and 2.4-fold respectively. Statistical testing using Kruskal-Wallis test revealed significant upregulation in expression of HSP70 of NP in response to static loading ($p = 0.047$) and dynamic loading ($p = 0.029$). Expression of HSP70 in AF did not change after static loading and was up-regulated very little with considerable variation after dynamic loading (Fig 4C). Expression of HSF1 (Fig 4B & 4D) did not show any significant difference between the loading types and control for both NP and AF.

Expression pattern of matrix remodeling genes were generally similar in NP and AF. The changes in expression of matrix anabolic genes in NP were not statistical significant. In AF, Kruskal-Wallis test demonstrates significant up-regulation in ACAN ($p = 0.010$) and COL2 ($p = 0.047$) after static loading compared to control. For gene expression of matrix catabolic genes under different loading types, ADAMTS4 and MMP13 in NP did not change much after dynamic loading (Fig 4K & 4M), whereas MMP3 expression (Fig 4L) was up-regulated by about 10-fold after static loading but was not statistical significant different. In AF tissue, ADAMTS4 expression was significantly down-regulated by about 9-fold in response to static loading ($p = 0.028$) but not dynamic loading (Fig 4N).

Fig 5 shows expressions of HSP70 and HSF1 at the protein level by immunofluorescence staining. Expression of HSP72, the inducible form of HSP70 was found in the cell nuclei and appeared as large patches (Fig 5A–5C). The patterns of HSP70 appear similar and did not change after different conditions but quantified signal intensity per nucleus is significantly higher after dynamic loading compared to control (Fig 5D, $p = 0.032$). For HSF1 (Fig 5E–5G), no large aggregates were observed in nuclei. However, signal intensity of HSF1 in the nuclei significantly increased after both static loading ($p = 0.019$) and dynamic loading ($p = 0.004$).

Repeated loading

Changes in expression of HSP70 in response to dynamic loading were transient (Fig 6A). Up-regulation continued to be observed 22 h after loading at day two ($p = 0.005$) compared to control. Similar trend was observed in HSF1 expression (Fig 6B). On the other hand, AF had a different expression trend from NP. Expression of HSP70 was only up-regulated at day 1 both right after loading and 22 h after loading but dropped to basal level at day 2 (Fig 6C) but without any significant differences.

Expression of ACAN appeared to be up-regulated right after each round of loading and down-regulated 22 h after loading in both tissues (Fig 6E & 6H). Expression of COL2 was up-

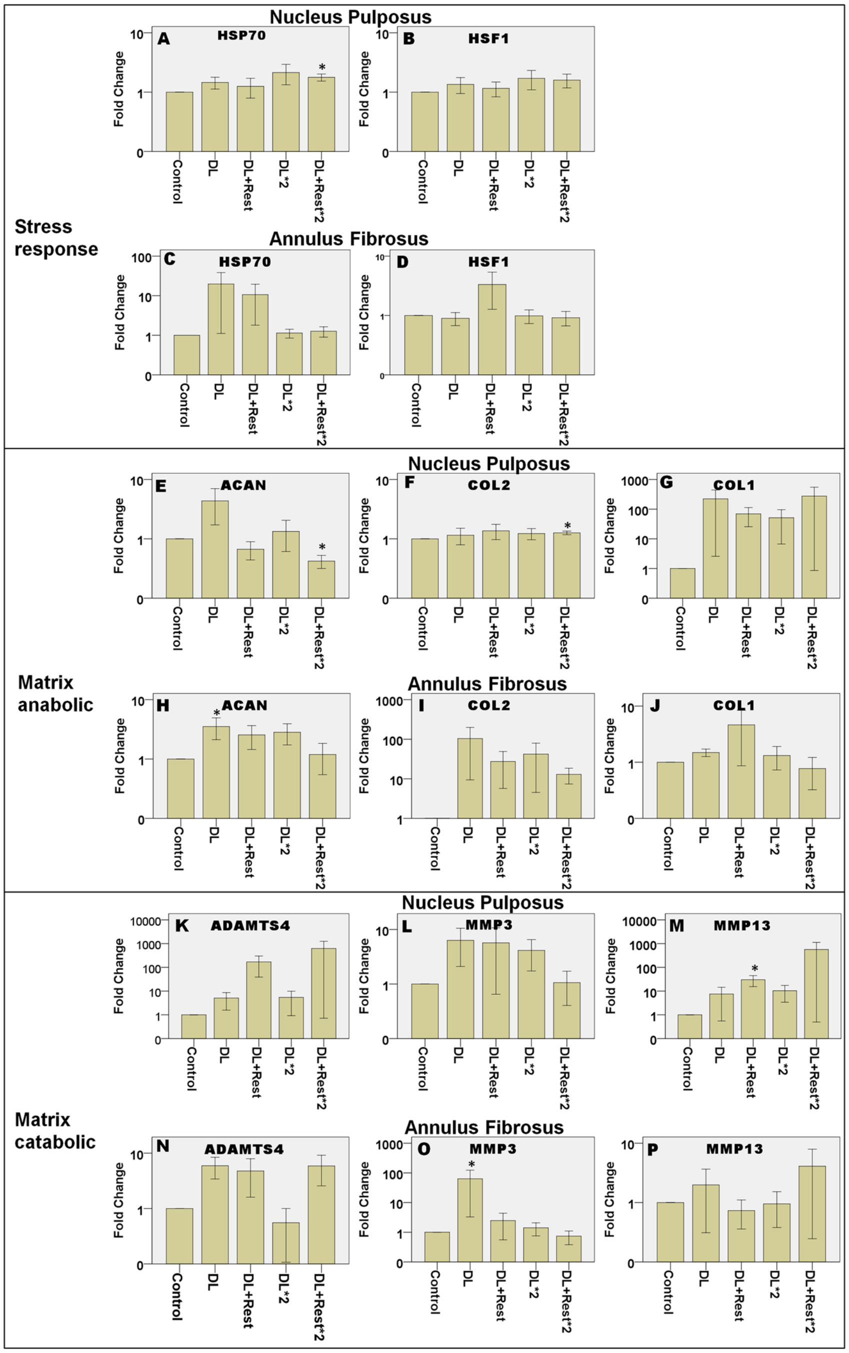

Fig 4. Relative gene expression after different loading types. Gene expressions are in log of fold change normalized to control without loading and represented in means ± SEM. SL: Static Loading, DL: Dynamic Loading. * = statistical significant difference with p < 0.05. N = 5 (from 3 animals) compared to control.

doi:10.1371/journal.pone.0161615.g004

regulated mildly in the DL + Rest*2 group of NP (Fig 6F, p = 0.005) compared to control. In contrast, expression of COL1 did not significantly change (Fig 6G & 6J). For matrix catabolic genes, expression of ADAMTS4 did not show significant changes (Fig 6K & 6N). Expression of MMP3 showed a similar trend in NP and AF, which increased significantly in response to dynamic loading in day 1 (p = 0.005) and decreased after resting in day 2 (Fig 6L & 6O).

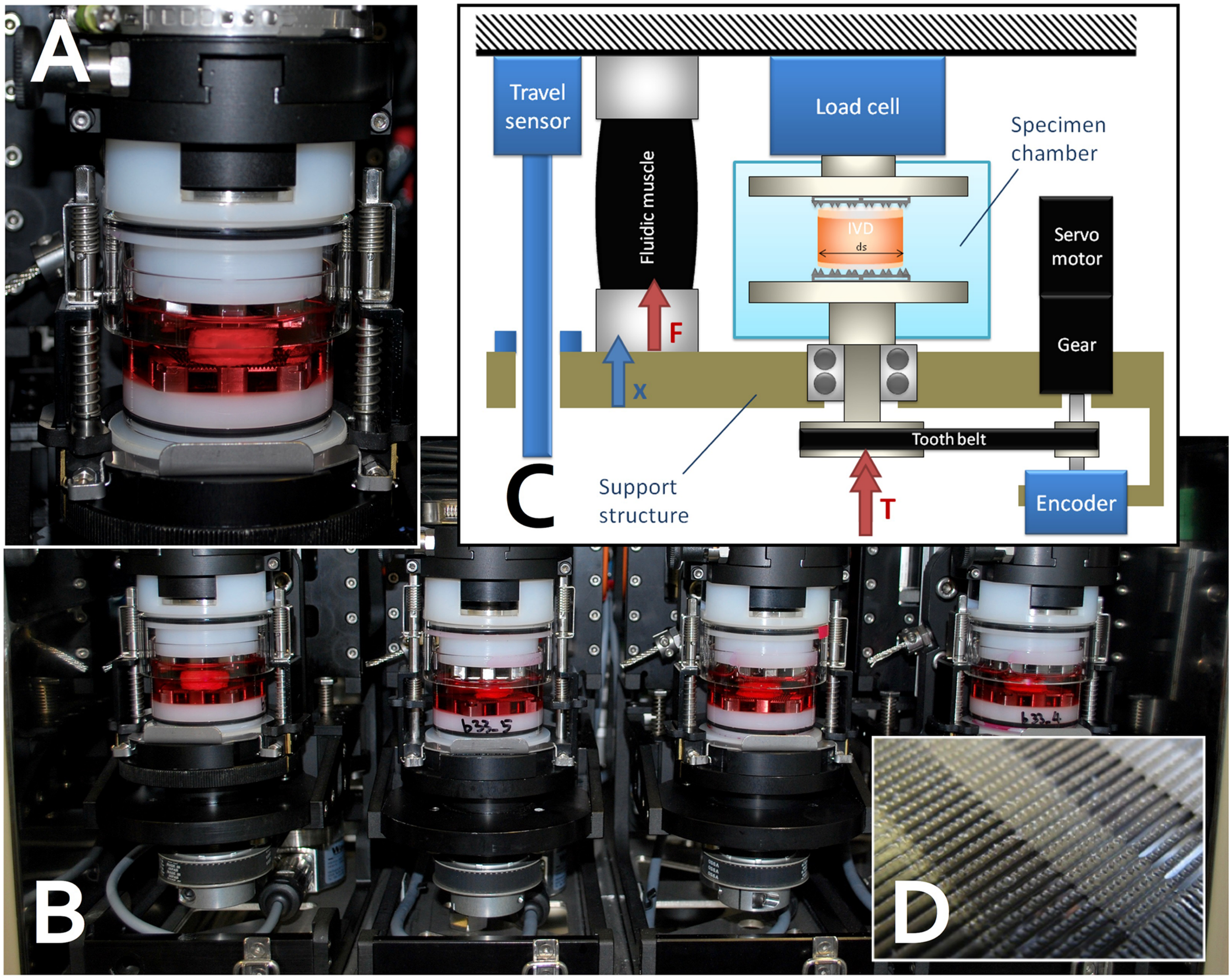

Fig 5. Expressions of HSP70 and HSF1 at the protein level shown by immunofluorescence staining. Representative images from nucleus pulposus after different conditions are shown. Tissues were immuno-labeled by antibody for HSP72, the inducible form of HSP70 (A-C) and HSF1 (E-G) and nuclei were labeled with DAPI. Signal intensity was quantified and presented as signal intensity per volume for each nucleus. 31 to 47 nuclei were analyzed for HSP70 and 23 to 42 nuclei were analyzed for HSF1 in each group. Control: without loading, SL: Static Loading, DL: Dynamic Loading. * = statistical significant difference with $p < 0.05$.

doi:10.1371/journal.pone.0161615.g005

Resting for 22 h after first round of loading upregulated MMP13 in NP by about 15-fold (Fig 6M, p = 0.005).

It is also notable that expressions of HSP70 (NP), HSF1 (NP), ACAN (NP and AF) and COL2 (AF) showed an oscillating trend with up-regulation right after loading and down-regulation after resting. In contrast, ADAMTS4 (NP) and MMP13 (NP) show opposite expression trend with up-regulation after resting.

Discussion

Studies have investigated different aspects on how the IVD cells respond towards loading but none of them explored the stress response of the IVD cells *in situ*. Expression of HSPs had been associated with disc degeneration, pathology and aging [23, 24]. The association was suggested to be related to mechanical and environment stress experienced by the cells but there is

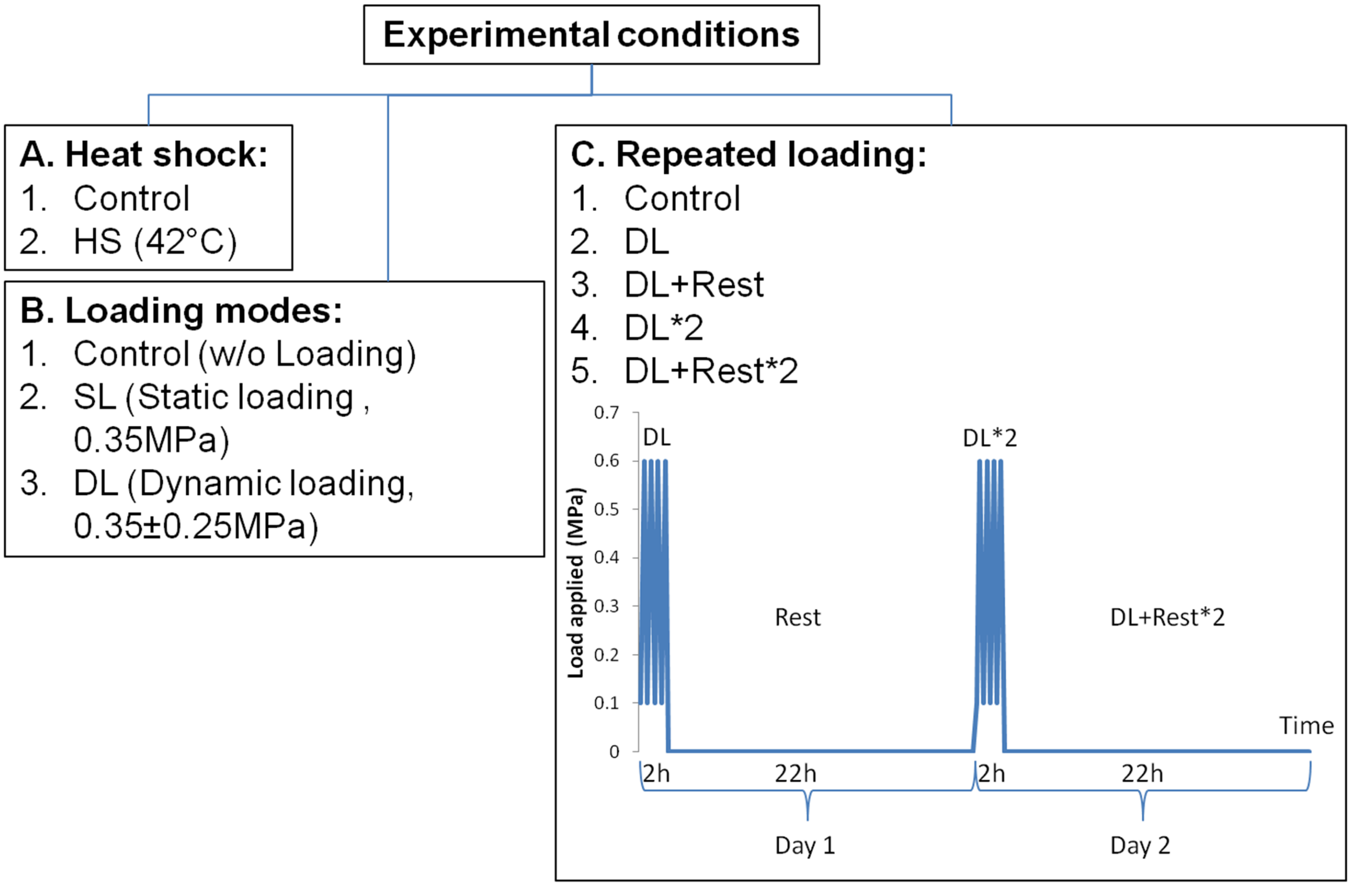

Fig 6. Relative gene expression after repeated dynamic loading and resting. Gene expressions are presented in log of fold change normalized to control without loading and represented in means ± SEM. DL: after dynamic loading, DL+Rest: after dynamic loading followed by resting, DL*2: after dynamic loading for 2 days, DL+Rest*2: after dynamic loading followed by resting for two days. N = 5. * = statistical significant difference with $p < 0.05$. N = 5 (from 5 animals) compared to control.

doi:10.1371/journal.pone.0161615.g006

no study that demonstrates the direct causal relationship of mechanical stress and expression of HSPs in IVD cells in their native ECM environment. Using organ culture, the results from the current study provide more evidence to this suggestion by demonstrating NPCs' expression of HSP70 in response to repetitive cyclic compressive loading. This finding suggests that healthy NPCs activate relevant stress response to cope with mechanical stress.

Throughout the study, disc cell's activity was maintained in all conditions, confirming that cell viability was not affected by short-term heat-shock nor compression. This is consistent with earlier results using similar magnitude of loading [30]. Both magnitudes were suggested to be in physiological range for large animal discs such as ovine [36, 37] and bovine discs [30].

Compressive loading appears to induce stress response in NPCs, even though only a relatively low physiological range magnitude was applied and cell activity was not altered. Heat-shock response was seen in NPCs under both static loading and dynamic loading but the fold change in response to compression is much lower than heat-shock, which was also reported in chondrocytes [35]. This effect was continually observed when the tissues were loaded for a second time under dynamic loading. The expression trend appears to oscillate with loading and resting. This further indicates that the major heat shock protein, HSP70 exclusively up-regulates after loading, while cells recover after rested. The other two heat shock proteins, HSP27 and HSP90 were only upregulated insignificantly in response to dynamic loading (S2 Fig). On

the other hand, up-regulation of HSP70 after loading was not observed in AFCs. This suggests that the deformation of the AFCs surrounding extra cellular matrix is smaller and the matrix shielded AFCs from mild mechanical stress under compression.

Mechanism and cell signaling pathway linking cells mechanical loading to cell fate is not fully understood. Reasons of mechanical loading caused stress response have been speculated previously. It was suggested that stress response is caused by cytoskeleton reorganization and protein synthesis due to mechanical loading [35, 38, 39]. Loading induced expression of HSP70 was further suggested to be leading to stress fiber formation. By using inhibitor, the study showed that inhibition of HSP70 would prevent HSP70 up-regulation and stress fibers formation after stretch. In contrast, disrupting formation of stress fibers had no effect on HSP70 up-regulation [13]. The authors hypothesized that cytoskeleton reorganization maybe an outcome rather than a cause of up-regulation of HSP70. In this study, substantial cytoskeleton reorganization was not observed with up-regulation of HSP70, supporting the hypothesis. Cytoskeleton reorganization may only happen with longer loading duration. On the other hand, global protein synthesis was demonstrated to be suppressed during loading and rebound after loading withdrawal [40]. This leads to a theory that stress response occurs as part of the chaperone activity due to protein synthesis [19, 35, 38, 41]. Besides, loading induced stress response is suspected to be a downstream event of the mechanotransduction pathway. Several mechanosensors were suggested to induce cellular stress responses. For example, stretch-activated ion channels were demonstrated to activate HSF1 and up-regulate HSP70 [42] while integrins were suggested to be coordinated with cellular stress response [43]. Expression of HSP70 involved in mechanical loading related chaperone activity and mechanotransduction pathway needs to be elucidated in future experiments.

The majority of the matrix remodeling genes, which include the matrix anabolic and catabolic genes were mildly up-regulated after static loading and dynamic loading. This finding is rather consistent with existing literature using bovine caudal disc model that most of the matrix remodeling genes were upregulated mildly for both tissues [30]. Significant up-regulation of ACAN and COL2 after dynamic loading and down-regulation of ADAMTS4 after static loading in AFCs suggest physiological range compression signals the AFCs to inhibit the matrix catabolic process and maintain the extracellular matrix. The results agree with existing literature that physiological range loading is more favorable for matrix maintenance of the disc [11, 12, 36]. Another study observed up-regulation of COL2 and ACAN in NPCs for a longer duration (> 7 days) of dynamic loading using caprine disc [36]. This was not observed in NP in this study, suggesting that the short-term loading (2 days) may not be enough to upregulate the matrix anabolic genes. However, dynamic loading appears to be more favorable to the matrix production in both NP and AF tissue.

Oscillating up- and down-regulation trends with loading and resting suggest that changes in expression of matrix remodeling genes in IVD cells were transient as demonstrated in earlier studies [44–46]. However, the trends of matrix anabolic and catabolic genes were different compared to the mentioned studies due to different models and experimental conditions. In this study, the anabolic and catabolic genes present opposite trend which suggest a different peak of mRNA expression may follow after loading.

Mechanism to link the expression of HSP70 to matrix maintenance has been demonstrated in cartilage and chondrocytes, where expression of HSP70 was shown to induce matrix production and prevent matrix degradation [47–49]. The observation in this study, up-regulation of HSP70 in NP without significant matrix remodeling, suggests that stress response is more likely to be induced before matrix remodeling and hence may be a downstream event of mechanotransduction pathway. The relationship between such cellular pathways and matrix remodeling mechanisms in response to loading needs further investigation.

Conclusion

This study investigated the stress response of the IVD towards mechanical loading. Compressive loading induced stress response was demonstrated in bovine caudal disc. Up-regulation of HSP70 was observed in the NPCs during compressive loading at physiological range, where cell activity was not changed and matrix remodeling was low.

Supporting Information

S1 Fig. Relative gene expression of stress response genes after heat-shock. Gene expressions are in log of fold change normalized to control without loading and represented in mean ±SEM. HS: Heat-Shock. * = statistical significant difference with $p<0.05$ compared to control. N = 5.
(TIF)

S2 Fig. Relative gene expression of HSP27 and HSP90 in response to different loading types. Gene expressions are in log of fold change normalized to control without loading and represented in means ± SEM. SL: Static Loading, DL: Dynamic Loading. N = 5 (from 3 animals) compared to control.
(TIF)

Author Contributions

Conceived and designed the experiments: WHC SCWC BG BPC.

Performed the experiments: WHC SCWC.

Analyzed the data: WHC SCWC BG BPC.

Contributed reagents/materials/analysis tools: BG BPC.

Wrote the paper: WHC SCWC BG BPC.

References

1. Luoma K, Riihimaki H, Luukkonen R, Raininko R, Viikari-Juntura E, Lamminen A. Low back pain in relation to lumbar disc degeneration. Spine. 2000; 25(4):487–92. Epub 2000/03/09. PMID: 10707396.
2. Battie MC, Videman T. Lumbar disc degeneration: epidemiology and genetics. The Journal of bone and joint surgery American volume. 2006; 88 Suppl 2:3–9. Epub 2006/04/06. doi: 10.2106/JBJS.E.01313 PMID: 16595435.
3. Chan D, Song Y, Sham P, Cheung KM. Genetics of disc degeneration. European spine journal: official publication of the European Spine Society, the European Spinal Deformity Society, and the European Section of the Cervical Spine Research Society. 2006; 15 Suppl 3:S317–25. Epub 2006/07/05. doi: 10.1007/s00586-006-0171-3 PMID: 16819621; PubMed Central PMCID: PMC2335375.
4. Kepler CK, Ponnappan RK, Tannoury CA, Risbud MV, Anderson DG. The molecular basis of intervertebral disc degeneration. The spine journal: official journal of the North American Spine Society. 2013; 13(3):318–30. Epub 2013/03/30. doi: 10.1016/j.spinee.2012.12.003 PMID: 23537454.
5. Urban JP, Roberts S. Degeneration of the intervertebral disc. Arthritis research & therapy. 2003; 5(3):120–30. Epub 2003/05/02. PMID: 12723977; PubMed Central PMCID: PMC165040.
6. Smith LJ, Nerurkar NL, Choi KS, Harfe BD, Elliott DM. Degeneration and regeneration of the intervertebral disc: lessons from development. Disease models & mechanisms. 2011; 4(1):31–41. Epub 2010/12/03. doi: 10.1242/dmm.006403 PMID: 21123625; PubMed Central PMCID: PMC3008962.
7. Buckwalter JA. Aging and degeneration of the human intervertebral disc. Spine. 1995; 20(11):1307–14. Epub 1995/06/01. PMID: 7660243.
8. Gruber HE, Hanley EN Jr. Analysis of aging and degeneration of the human intervertebral disc. Comparison of surgical specimens with normal controls. Spine. 1998; 23(7):751–7. Epub 1998/05/01. PMID: 9563104.

9. Lotz JC, Colliou OK, Chin JR, Duncan NA, Liebenberg E. Compression-induced degeneration of the intervertebral disc: an in vivo mouse model and finite-element study. Spine. 1998; 23(23):2493–506. Epub 1998/12/17. PMID: 9854748.

10. Lipson SJ, Muir H. Experimental intervertebral disc degeneration: morphologic and proteoglycan changes over time. Arthritis and rheumatism. 1981; 24(1):12–21. Epub 1981/01/01. PMID: 7470167.

11. Setton LA, Chen J. Mechanobiology of the intervertebral disc and relevance to disc degeneration. The Journal of bone and joint surgery American volume. 2006; 88 Suppl 2:52–7. Epub 2006/04/06. doi: 10.2106/JBJS.F.00001 PMID: 16595444.

12. Chan SC, Ferguson SJ, Gantenbein-Ritter B. The effects of dynamic loading on the intervertebral disc. European spine journal: official publication of the European Spine Society, the European Spinal Deformity Society, and the European Section of the Cervical Spine Research Society. 2011; 20(11):1796–812. Epub 2011/05/05. doi: 10.1007/s00586-011-1827-1 PMID: 21541667; PubMed Central PMCID: PMC3207351.

13. Luo SS, Sugimoto K, Fujii S, Takemasa T, Fu SB, Yamashita K. Role of heat shock protein 70 in induction of stress fiber formation in rat arterial endothelial cells in response to stretch stress. Acta histochemica et cytochemica. 2007; 40(1):9–17. Epub 2007/03/22. doi: 10.1267/ahc.06011 PMID: 17375204; PubMed Central PMCID: PMC1828078.

14. Kippenberger S, Bernd A, Loitsch S, Muller J, Guschel M, Kaufmann R. Cyclic stretch up-regulates proliferation and heat shock protein 90 expression in human melanocytes. Pigment cell research / sponsored by the European Society for Pigment Cell Research and the International Pigment Cell Society. 1999; 12(4):246–51. Epub 1999/08/24. PMID: 10454292.

15. Galvin DJ, Watson RW, Gillespie JI, Brady H, Fitzpatrick JM. Mechanical stretch regulates cell survival in human bladder smooth muscle cells in vitro. American journal of physiology Renal physiology. 2002; 283(6):F1192–9. Epub 2002/10/22. doi: 10.1152/ajprenal.00168.2002 PMID: 12388384.

16. Xu Q, Schett G, Li C, Hu Y, Wick G. Mechanical stress-induced heat shock protein 70 expression in vascular smooth muscle cells is regulated by Rac and Ras small G proteins but not mitogen-activated protein kinases. Circulation research. 2000; 86(11):1122–8. Epub 2000/06/13. PMID: 10850962.

17. Enokiya Y, Hashimoto S, Muramatsu T, Jung HS, Tazaki M, Inoue T, et al. Effect of stretching stress on gene transcription related to early-phase differentiation in rat periodontal ligament cells. The Bulletin of Tokyo Dental College. 2010; 51(3):129–37. Epub 2010/09/30. PMID: 20877159.

18. Luna C, Li G, Liton PB, Epstein DL, Gonzalez P. Alterations in gene expression induced by cyclic mechanical stress in trabecular meshwork cells. Molecular vision. 2009; 15:534–44. Epub 2009/03/13. PMID: 19279691; PubMed Central PMCID: PMC2654047.

19. Jagodzinski M, Hankemeier S, van Griensven M, Bosch U, Krettek C, Zeichen J. Influence of cyclic mechanical strain and heat of human tendon fibroblasts on HSP-72. European journal of applied physiology. 2006; 96(3):249–56. Epub 2005/11/02. doi: 10.1007/s00421-005-0071-y PMID: 16261388.

20. Fitzgerald JB, Jin M, Dean D, Wood DJ, Zheng MH, Grodzinsky AJ. Mechanical compression of cartilage explants induces multiple time-dependent gene expression patterns and involves intracellular calcium and cyclic AMP. The Journal of biological chemistry. 2004; 279(19):19502–11. Epub 2004/02/13. doi: 10.1074/jbc.M400437200 PMID: 14960571.

21. Kaarniranta K, Holmberg CI, Lammi MJ, Eriksson JE, Sistonen L, Helminen HJ. Primary chondrocytes resist hydrostatic pressure-induced stress while primary synovial cells and fibroblasts show modified Hsp70 response. Osteoarthritis and cartilage / OARS, Osteoarthritis Research Society. 2001; 9(1):7–13. Epub 2001/02/17. doi: 10.1053/joca.2000.0354 PMID: 11178942.

22. Nakamura S, Arai Y, Takahashi KA, Terauchi R, Ohashi S, Mazda O, et al. Hydrostatic pressure induces apoptosis of chondrocytes cultured in alginate beads. Journal of orthopaedic research: official publication of the Orthopaedic Research Society. 2006; 24(4):733–9. Epub 2006/03/04. doi: 10.1002/jor.20077 PMID: 16514637.

23. Takao T, Iwaki T. A comparative study of localization of heat shock protein 27 and heat shock protein 72 in the developmental and degenerative intervertebral discs. Spine. 2002; 27(4):361–8. Epub 2002/02/13. PMID: 11840100.

24. Sharp CA, Roberts S, Evans H, Brown SJ. Disc cell clusters in pathological human intervertebral discs are associated with increased stress protein immunostaining. European spine journal: official publication of the European Spine Society, the European Spinal Deformity Society, and the European Section of the Cervical Spine Research Society. 2009; 18(11):1587–94. Epub 2009/06/12. doi: 10.1007/s00586-009-1053-2 PMID: 19517141; PubMed Central PMCID: PMC2899395.

25. Lee CR, Iatridis JC, Poveda L, Alini M. In vitro organ culture of the bovine intervertebral disc: effects of vertebral endplate and potential for mechanobiology studies. Spine. 2006; 31(5):515–22. Epub 2006/03/02. doi: 10.1097/01.brs.0000201302.59050.72 PMID: 16508544.

26. Gantenbein B, Illien-Junger S, Chan SC, Walser J, Haglund L, Ferguson SJ, et al. Organ culture bioreactors—platforms to study human intervertebral disc degeneration and regenerative therapy. Current stem cell research & therapy. 2015; 10(4):339–52. Epub 2015/03/13. PMID: 25764196; PubMed Central PMCID: PMC4437861.

27. Korecki CL, MacLean JJ, Iatridis JC. Dynamic compression effects on intervertebral disc mechanics and biology. Spine. 2008; 33(13):1403–9. Epub 2008/06/04. doi: 10.1097/BRS.0b013e318175cae7 PMID: 18520935; PubMed Central PMCID: PMC2566854.

28. Schmocker A, Khoushabi A, Frauchiger DA, Gantenbein B, Schizas C, Moser C, et al. A photopolymerized composite hydrogel and surgical implanting tool for a nucleus pulposus replacement. Biomaterials. 2016; 88:110–9. Epub 2016/03/16. doi: 10.1016/j.biomaterials.2016.02.015 PMID: 26976264.

29. Chan SC, Ferguson SJ, Wuertz K, Gantenbein-Ritter B. Biological response of the intervertebral disc to repetitive short-term cyclic torsion. Spine. 2011; 36(24):2021–30. Epub 2011/02/24. doi: 10.1097/BRS.0b013e318203aea5 PMID: 21343864.

30. Chan SC, Walser J, Kappeli P, Shamsollahi MJ, Ferguson SJ, Gantenbein-Ritter B. Region specific response of intervertebral disc cells to complex dynamic loading: an organ culture study using a dynamic torsion-compression bioreactor. PloS one. 2013; 8(8):e72489. Epub 2013/09/10. doi: 10.1371/journal.pone.0072489 PMID: 24013824; PubMed Central PMCID: PMC3755972.

31. Chan SC, Gantenbein-Ritter B. Preparation of intact bovine tail intervertebral discs for organ culture. Journal of visualized experiments: JoVE. 2012;(60:). Epub 2012/02/15. doi: 10.3791/3490 PMID: 22330901; PubMed Central PMCID: PMC3369634.

32. Walser J, Ferguson SJ, Gantenbein-Ritter B. Design of a Mechanical Loading Device to Culture Intact Bovine Spinal Motion Segments under Multiaxial Motion. Replacing Animal Models: A Practical Guide to Creating and Using Culture-based Biomimetic Alternatives. 2012: 89.

33. Xiao J, Zhang Y, Wang J, Yu W, Wang W, Ma X. Monitoring of cell viability and proliferation in hydrogel-encapsulated system by resazurin assay. Applied biochemistry and biotechnology. 2010; 162 (7):1996–2007. Epub 2010/05/04. doi: 10.1007/s12010-010-8975-3 PMID: 20437208.

34. Morimoto R. Heat-Shock Response. Encyclopedia of Molecular Biology: John Wiley & Sons, Inc.; 2002.

35. Kaarniranta K, Elo M, Sironen R, Lammi MJ, Goldring MB, Eriksson JE, et al. Hsp70 accumulation in chondrocytic cells exposed to high continuous hydrostatic pressure coincides with mRNA stabilization rather than transcriptional activation. Proceedings of the National Academy of Sciences of the United States of America. 1998; 95(5):2319–24. Epub 1998/04/16. PMID: 9482883; PubMed Central PMCID: PMC19331.

36. Paul CP, Zuiderbaan HA, Zandieh Doulabi B, van der Veen AJ, van de Ven PM, Smit TH, et al. Simulated-physiological loading conditions preserve biological and mechanical properties of caprine lumbar intervertebral discs in ex vivo culture. PloS one. 2012; 7(3):e33147. Epub 2012/03/20. doi: 10.1371/journal.pone.0033147 PMID: 22427972; PubMed Central PMCID: PMC3302815.

37. Illien-Junger S, Gantenbein-Ritter B, Grad S, Lezuo P, Ferguson SJ, Alini M, et al. The combined effects of limited nutrition and high-frequency loading on intervertebral discs with endplates. Spine. 2010; 35(19):1744–52. Epub 2010/04/17. doi: 10.1097/BRS.0b013e3181c48019 PMID: 20395884.

38. Takahashi K, Kubo T, Kobayashi K, Imanishi J, Takigawa M, Arai Y, et al. Hydrostatic pressure influences mRNA expression of transforming growth factor-beta 1 and heat shock protein 70 in chondrocyte-like cell line. Journal of orthopaedic research: official publication of the Orthopaedic Research Society. 1997; 15(1):150–8. Epub 1997/01/01. doi: 10.1002/jor.1100150122 PMID: 9066540.

39. Haskin CL, Athanasiou KA, Klebe R, Cameron IL. A heat-shock-like response with cytoskeletal disruption occurs following hydrostatic pressure in MG-63 osteosarcoma cells. Biochemistry and cell biology = Biochimie et biologie cellulaire. 1993; 71(7–8):361–71. Epub 1993/07/01. 7510113. PMID: 7510113

40. Lomas C, Tang XD, Chanalaris A, Saklatvala J, Vincent TL. Cyclic mechanical load causes global translational arrest in articular chondrocytes: a process which is partially dependent upon PKR phosphorylation. European cells & materials. 2011; 22:178–89. Epub 2011/09/21. PMID: 21932194.

41. Kuperman DI, Freyaldenhoven TE, Schmued LC, Ali SF. Methamphetamine-induced hyperthermia in mice: examination of dopamine depletion and heat-shock protein induction. Brain research. 1997; 771 (2):221–7. Epub 1997/12/24. PMID: 9401742.

42. Chang J, Wasser JS, Cornelussen RN, Knowlton AA. Activation of heat-shock factor by stretch-activated channels in rat hearts. Circulation. 2001; 104(2):209–14. Epub 2001/07/12. PMID: 11447088.

43. Stupack DG, Cheresh DA. Get a ligand, get a life: integrins, signaling and cell survival. Journal of cell science. 2002; 115(Pt 19):3729–38. Epub 2002/09/18. PMID: 12235283.

44. MacLean JJ, Roughley PJ, Monsey RD, Alini M, Iatridis JC. In vivo intervertebral disc remodeling: kinetics of mRNA expression in response to a single loading event. Journal of orthopaedic research: official

publication of the Orthopaedic Research Society. 2008; 26(5):579–88. Epub 2008/01/08. doi: 10.1002/jor.20560 PMID: 18176944; PubMed Central PMCID: PMC2570195.

45. Iatridis JC, MacLean JJ, Roughley PJ, Alini M. Effects of mechanical loading on intervertebral disc metabolism in vivo. The Journal of bone and joint surgery American volume. 2006; 88 Suppl 2:41–6. Epub 2006/04/06. doi: 10.2106/JBJS.E.01407 PMID: 16595442; PubMed Central PMCID: PMC3697474.

46. McCann MR, Patel P, Beaucage KL, Xiao Y, Bacher C, Siqueira WL, et al. Acute vibration induces transient expression of anabolic genes in the murine intervertebral disc. Arthritis and rheumatism. 2013; 65 (7):1853–64. Epub 2013/05/11. doi: 10.1002/art.37979 PMID: 23661269.

47. Arai Y, Kubo T, Kobayashi K, Takeshita K, Takahashi K, Ikeda T, et al. Adenovirus vector-mediated gene transduction to chondrocytes: in vitro evaluation of therapeutic efficacy of transforming growth factor-beta 1 and heat shock protein 70 gene transduction. The Journal of rheumatology. 1997; 24 (9):1787–95. Epub 1997/09/18. PMID: 9292805.

48. Tsuchida S, Arai Y, Takahashi KA, Kishida T, Terauchi R, Honjo K, et al. HIF-1alpha-induced HSP70 regulates anabolic responses in articular chondrocytes under hypoxic conditions. Journal of orthopaedic research: official publication of the Orthopaedic Research Society. 2014; 32(8):975–80. Epub 2014/03/29. doi: 10.1002/jor.22623 PMID: 24677016.

49. Fujita S, Arai Y, Nakagawa S, Takahashi KA, Terauchi R, Inoue A, et al. Combined microwave irradiation and intraarticular glutamine administration-induced HSP70 expression therapy prevents cartilage degradation in a rat osteoarthritis model. Journal of orthopaedic research: official publication of the Orthopaedic Research Society. 2012; 30(3):401–7. Epub 2011/08/20. doi: 10.1002/jor.21535 PMID: 21853458.
